# Fruit- and Vegetable-Derived Exosome-like Nanovesicles: A Review

**DOI:** 10.3390/foods15183194

**Published:** 2026-09-09

**Authors:** Yi Nie, Na Li, Hong-Tao Zhu, Ying-Jun Zhang

**Affiliations:** 1 State Key Laboratory of Phytochemistry & Natural Medicines (CAS), Kunming Institute of Botany, Chinese Academy of Sciences, Kunming 650201, China; nieyi@mail.kib.ac.cn; 2 University of Chinese Academy of Sciences, Beijing 100049, China; 3 Key Laboratory of Phytochemistry and Plant Resources in West China, Kunming Institute of Botany, Chinese Academy of Sciences, Kunming 650201, China; lina1@mail.kib.ac.cn (N.L.); zhuhongtao@mail.kib.ac.cn (H.-T.Z.)

**Keywords:** fruit- and vegetable-derived exosome-like nanovesicles, isolation and characterization, physiological activities, application potential, food sector

## Abstract

Fruit- and vegetable-derived exosome-like nanovesicles (FV-ELNs) are nanovesicles characterized by a lipid bilayer membrane that encloses a diverse array of biologically active constituents, including proteins, lipids, nucleic acids, and small-molecule bioactive compounds. FV-ELNs have attracted considerable research interest in recent years due to their favorable biocompatibility and natural origin. The bioactive cargo encapsulated within these vesicles confers a range of pharmacological activities, encompassing anti-inflammatory, gut microbiota-modulating, and antioxidant effects, suggesting their potential for applications in food preservation, functional food development, and bioactive compound delivery. This review presents the current methods for isolation and characterization of FV-ELNs, their compositional features, and bioactivities, as well as the potential for food-industry applications. Moreover, the challenges and future directions are also addressed, offering an updated perspective on their role as natural complements to synthetic agents in functional foods and dietary interventions.

## 1. Introduction

**Figure 1:**
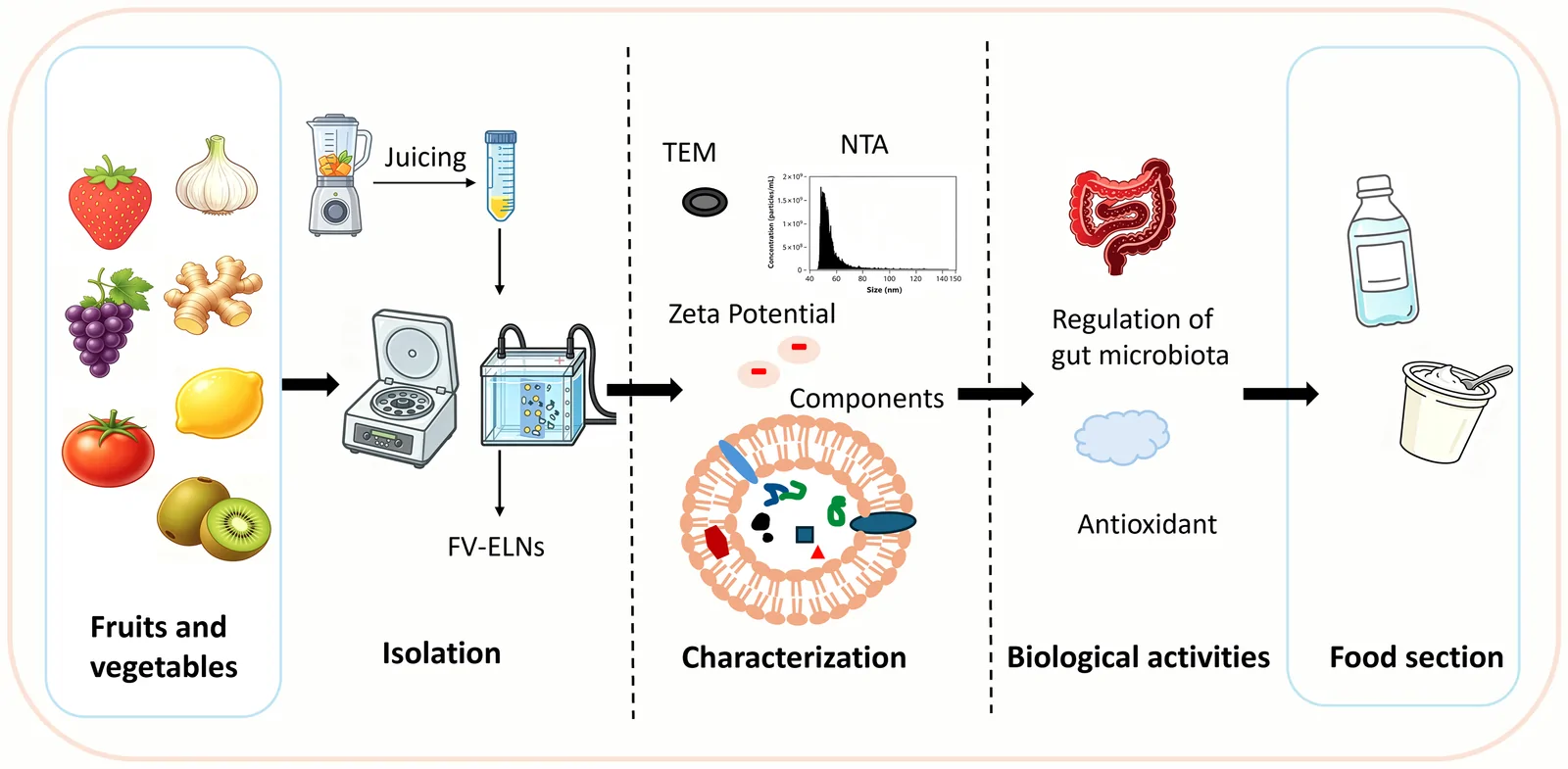
FV-ELNs: sources, isolation, characterization, biological functions, and food application prospects.

Exosomes are extracellular vesicles (EVs) with a bilayer membrane structure, produced commonly by both mammalian and plant cells. They not only participate in immunogenic responses, but also serve as key mediators of intercellular communication, playing a regulatory role in various diseases [1]. Due to the complex structures and inherent heterogeneity of plant tissues, isolates obtained from plants through physical tissue disruption using current extraction methods often contain other microvesicles and impurities, and are therefore collectively referred to as plant-derived exosome-like nanovesicles (PELNs), which share structural features and biological activities similar to those of mammalian counterparts [2]. The PELNs exhibit a broad range of physiological activities, including antioxidant and anti-inflammatory effects, suggesting their potential for both functional food development and use as delivery vehicles for food-derived bioactive compounds [3]. Among various plant sources, fruit- and vegetable-derived exosome-like nanovesicles (FV-ELNs) are characterized by relatively wide availability, low immunogenicity, and potential for cost-effective scalability, and have thus attracted considerable attention [4]. Compared with conventional plant extracts, FV-ELNs offer certain advantages: for instance, up to 1 × 10^12^ nanoparticles can be extracted from each gram of ginger tissue [5], and ginger-derived exosome-like nanovesicles (GELNs) have demonstrated superior therapeutic efficacy against periodontitis compared with ginger juice [6]. To date, FV-ELNs have been successfully isolated from *citrus* fruits, berries, root vegetables, leafy vegetables, and gourds and solanaceous vegetables. However, existing research has predominantly focused on medical applications, while comprehensive investigations within the food domain remain relatively fragmented. In this context, the present review consolidates recent literature, providing an overview of isolation methodologies, characterization techniques, compositional profiles, and biological activities of FV-ELNs, with emphasis on their application potential as natural antioxidants, gut health-modulating functional ingredients, and bioactive compound delivery carriers in the food industry. In addition, existing challenges and future prospects are highlighted. This review aims to provide a theoretical foundation for the in-depth investigation and industrial application of FV-ELNs in the food sector. The overall framework of this review—covering sources, isolation, characterization, biological functions, and food-related applications—is schematically illustrated in Figure 1.

## 2. Isolation of FV-ELNs

**Figure 2:**
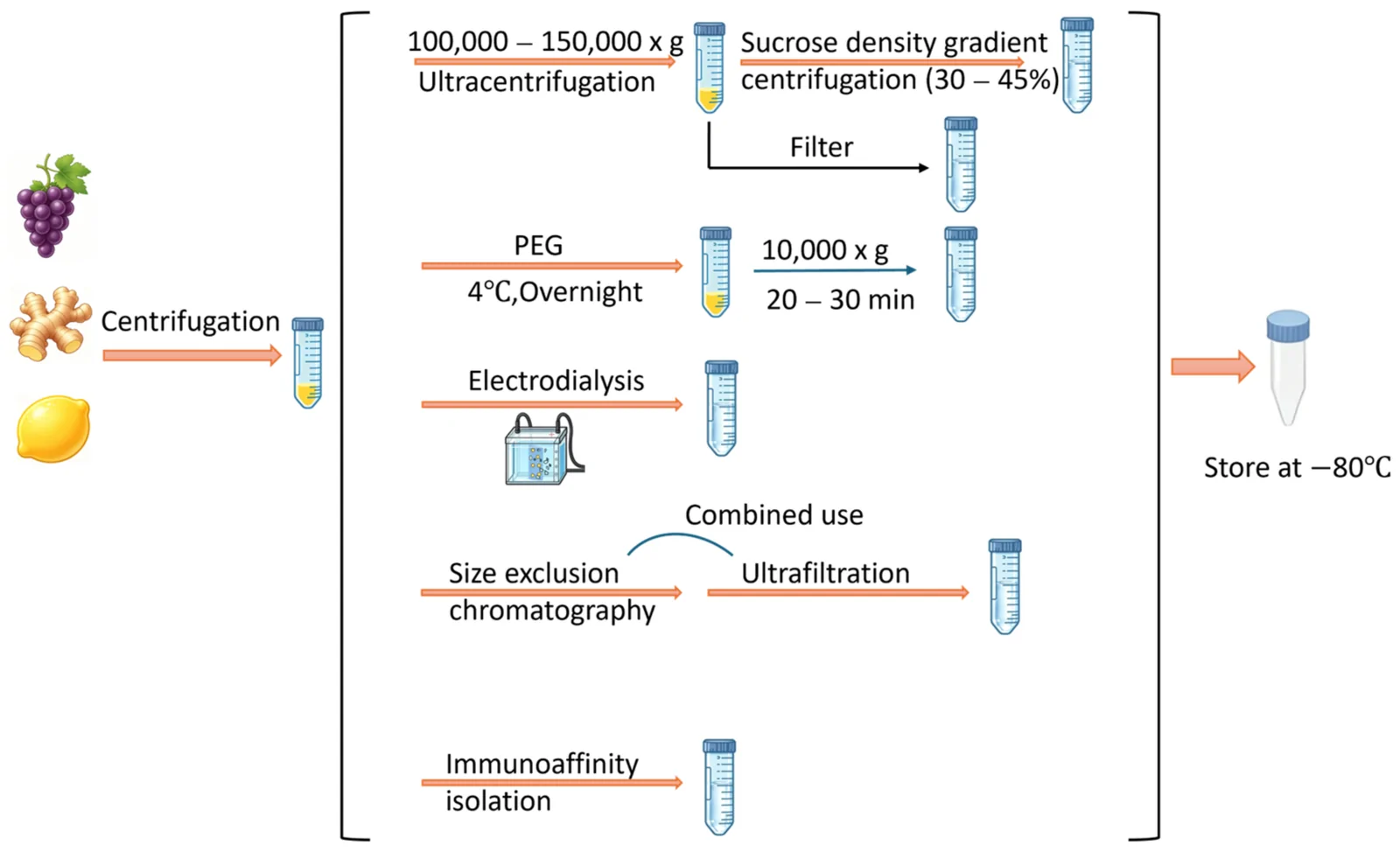
Isolation methods of FV-ELNs.

The isolation of FV-ELNs is primarily adapted from methods established for the isolation of mammalian exosomes. However, plant tissues possess unique structural features including cell walls and vacuoles, along with an abundance of intracellular metabolites. These interfering factors render the direct application of mammalian exosome isolation protocols inadequate for obtaining FV-ELNs with both high purity and high bioactivity. Consequently, optimization of isolation techniques is imperative, with full consideration of the structural characteristics specific to plant tissues. In the pre-treatment stage, strategies such as juicing, mild homogenization, and enzyme-assisted digestion are employed to disrupt the cell wall barrier and minimize tissue debris and contaminating residues. During centrifugation, appropriate parameters must be carefully selected to ensure optimal separation. Currently, the most commonly employed methods include ultracentrifugation, sucrose density gradient centrifugation, and polymer precipitation. A comprehensive comparison of the advantages, limitations, and applicable scenarios of each method is summarized in Table 1. The operational principles and procedural workflows of these isolation methods are schematically depicted in Figure 2.

### 2.1. Ultracentrifugation

Ultracentrifugation is based on the differential sedimentation rates of sample components under varying centrifugal forces, enabling the isolation of FV-ELNs through gradient centrifugation techniques [7]. This method offers the advantages of simple operation and well-established protocols. However, it suffers from several inherent limitations. First, the multi-step gradient centrifugation process is time-consuming. Second, the limited capacity of centrifuge tubes restricts the sample volume that can be processed per run, making it difficult to meet the requirements for large-scale isolation of FV-ELNs. Finally, ultracentrifugation relies solely on differences in particle size and density for separation, lacking specific targeting or affinity-based enrichment capabilities. Consequently, the resulting FV-ELNs are susceptible to contamination and generally exhibit suboptimal purity.

### 2.2. Sucrose Density Gradient Centrifugation

Sucrose density gradient centrifugation is a modified version of ultracentrifugation that enables the isolation of FV-ELNs with substantially higher purity. This method exploits the inherent density differences between FV-ELNs and impurities. Under centrifugal force, various components migrate along the sucrose density gradient and sediment to their respective isopycnic zones, thereby achieving efficient and precise separation of FV-ELNs from contaminants [8].

### 2.3. Polymer Precipitation Method

The polymer precipitation method employs polyethylene glycol (PEG) as a co-precipitant, which reduces the solubility of FV-ELNs and induces their precipitation, allowing the recovery of FV-ELNs via low-speed centrifugation [9]. This approach is characterized by its operational simplicity, eliminating the need for sophisticated equipment and time-consuming procedures, while also accommodating large-scale sample processing. However, PEG decreases the solubility of most particles in the solution, causing proteins and other contaminants to co-precipitate with FV-ELNs, thereby resulting in relatively low final purity. The PEG-dextran two-phase partitioning system represents an optimized variant of the polymer precipitation method. Under centrifugal force, FV-ELNs tend to partition preferentially into the dextran phase, while proteins and other impurities remain predominantly in the PEG phase. This selective partitioning effect effectively reduces contaminant carryover and significantly improves the purity of FV-ELNs [10].

### 2.4. Electrodialysis

Electrodialysis is an isolation technique for FV-ELNs that integrates the principles of electrophoretic migration and dialysis retention, based on the size differences between FV-ELNs and contaminating molecules [11]. Under the driving force of an external electric field, small-molecule impurities such as proteins migrate through the membrane pores, while FV-ELNs are efficiently retained within the dialysis bag, thereby achieving selective separation. This method is cost-effective, requires no complex or expensive equipment, and offers high separation efficiency, rendering it a rapid and efficient approach for obtaining FV-ELNs.

### 2.5. Ultrafiltration

Ultrafiltration employs membranes with specific pore sizes to achieve separation: under pressure or centrifugal force, small-molecule impurities pass through the membrane pores while FV-ELNs are retained [12]. This method offers rapid processing and is amenable to scale-up for large sample volumes. However, ultrafiltration membranes are prone to fouling and clogging, and the squeezing effect generated during the process may potentially alter the structural integrity of FV-ELNs. To address these issues, studies have demonstrated that ultrafiltration combined with anion exchange chromatography enables efficient isolation of FV-ELNs under mild conditions. This integrated approach not only preserves the morphological integrity and biological activity of FV-ELNs but also improves sample purity and particle size uniformity [13].

### 2.6. Size Exclusion Chromatography

Size exclusion chromatography (SEC) is a separation technique based on differences in molecular size. Larger particles are excluded from the gel pores and thus elute more rapidly, while smaller particles are retained within the pores and elute at a slower rate [14], thereby achieving effective separation. SEC can be performed using gravity flow or low-speed centrifugation, which preserves the biological functionality and structural integrity of FV-ELNs. This method is often combined with ultrafiltration to obtain FV-ELNs with enhanced purity and higher bioactivity [15].

### 2.7. Immunoaffinity Isolation

Immunoaffinity isolation relies on specific binding between surface markers on FV-ELNs and corresponding antibodies conjugated to magnetic beads or affinity columns, enabling the capture and recovery of FV-ELNs through antigen–antibody specific interactions. This method offers high selectivity and yields products of exceptional purity, making it suitable for the isolation of specific vesicle subpopulations [16]. However, its application remains limited due to the high cost and the requirement for well-characterized surface markers, which are not yet fully established for most plant-derived vesicles.

In summary, no single method currently available can fulfill all requirements for FV-ELN isolation in terms of yield, purity, bioactivity preservation, and scalability. The choice of isolation strategy should therefore be tailored to the specific research objectives and downstream applications. For instance, polymer precipitation offers a practical option when high yield is the primary concern, whereas density gradient centrifugation and size exclusion chromatography are preferred when high purity is required. Ultracentrifugation, despite its limitations, may serve as a compromise when both yield and purity are of moderate concern. Notably, the combination of multiple methods, such as ultrafiltration coupled with size exclusion chromatography or anion exchange chromatography, has been shown to synergistically improve both purity and bioactivity, representing a promising direction for method optimization.

## 3. Physical Characterization

Physical characterization of FV-ELNs is essential for elucidating the structural basis underlying their biological functions and for ensuring the stability of their practical applications.

### 3.1. Morphological Characterization

FV-ELNs typically exhibit spherical or cup-shaped structures. Electron microscopy techniques are routinely employed for morphological characterization, including transmission electron microscopy (TEM), atomic force microscopy (AFM), scanning electron microscopy (SEM), and cryogenic electron microscopy (Cryo-EM). The advantages and limitations of these four morphological characterization techniques are summarized in Table 2.

Cryo-EM is the most reliable technique for directly visualizing lipid bilayer membranes, as it allows observation of the vesicular bilayer structure in its native hydrated state, avoiding the cup-shaped artifacts introduced by negative staining. In contrast, conventional negative-stain TEM can only visualize particle morphology and cannot conclusively confirm the presence of a phospholipid bilayer. SEM and AFM provide only surface topographic information and cannot assess membrane structure.

### 3.2. Particle Size, Zeta Potential, Purity and Yield

FV-ELNs typically exhibit zeta potential values ranging from near-neutral to −50 mV. In general, a higher absolute value of zeta potential corresponds to improved suspension stability of the vesicles. Notably, FV-ELNs isolated from the same plant source can display distinct particle sizes and zeta potential profiles depending on the extraction method employed. Isolation protocols alongside physical characterization data for FV-ELNs derived from various plant sources are compiled in Table 3.

Dynamic light scattering (DLS) and nanoparticle tracking analysis (NTA) are the two most widely employed techniques for the characterization of FV-ELN particle size. DLS instruments are typically equipped with an integrated electrophoretic light scattering (ELS) module for zeta potential measurement. DLS determines particle size by detecting time-dependent fluctuations in light intensity. This method offers the advantages of simple operation and rapid detection. However, it is highly sensitive to the presence of large particulate impurities in the sample, which can readily interfere with the measurement and lead to deviations, rendering DLS more suitable for monodisperse systems. NTA, in contrast, tracks individual particles based on light scattering and Brownian motion to determine vesicle size and concentration. This technique enables real-time capture of the Brownian motion trajectories of individual FV-ELNs and provides quantitative statistical analysis. NTA effectively minimizes interference from large particles, not only improving the accuracy of size measurement but also yielding concentration data, making it better suited for the characterization of heterogeneous and complex samples. The bicinchoninic acid assay is employed for the quantitative determination of FV-ELN protein concentration. When combined with particle concentration data, this allows calculation of purity (particle number per unit mass of protein) and yield (particle number per unit mass of raw material). It is noted, however, that the particle-to-protein ratio is at best a limited proxy for purity, as it does not distinguish vesicle-associated proteins from co-isolated soluble proteins or protein aggregates. As emphasized in the MISEV2023 guidelines, this ratio should not be used as a sole indicator of purity; instead, complementary approaches are required for a comprehensive assessment [17]. In current practice, lipid bilayer staining with membrane dyes such as FM4-64 is commonly used to verify vesicle membrane integrity, and Western blotting against plant-derived negative markers, including RbcL, a marker for chloroplast contamination, and calnexin, a marker for endoplasmic reticulum contamination, is employed to exclude co-isolated cellular debris. Together with the particle-to-protein ratio, these methods enable a more rigorous evaluation of both the purity and structural integrity of FV-ELNs.

**Table 3:** Isolation methods and physical characterization of different plants.

Plant Source	Isolation Method	Size (nm)	Zeta Potential (mV)	Morphology
Garlic [18]	Polymer precipitation + ultracentrifugation	100–300 (avg. 160.61)	−7.8	Spherical
Blueberry [19]	Polymer precipitation	150–250 (avg. 189.62)	−2.52	Spherical
Tomato [20]	Sucrose density gradient centrifugation	120–140	−20 to −30	Spherical
Tomato [21]	Ultrafiltration + Polymer precipitation	30–200	—	Round
Grapefruit [22]	Ultracentrifugation	86–125	−10	Spherical
Grape [23]	Sucrose density gradient centrifugation	avg. 380.5 ± 37.47	avg. −26.3 ± 8.14	Round
Grape [24]	Ultracentrifugation + Sucrose density gradient centrifugation	avg. 163.4	—	Cup-shaped
Ginger [6]	Ultracentrifugation	avg. 161.2	−35.7	Spherical
Ginger [25]	Ultracentrifugation + Sucrose density gradient centrifugation	avg. 188.5	−15.5	Spherical
Broccoli [26]	Ultracentrifugation + Ultrafiltration	18.3–118.2 (avg. 32.4)	−39.2 to −2.62 (avg. −17.1)	Spherical
Carrot [27]	Ultrafiltration + Size-exclusion chromatography	avg. 143.9	avg. −10.2	Spherical
Strawberry [28]	Ultracentrifugation	30–191	—	Spherical
Apple [29]	Ultracentrifugation	avg. 115–120	—	Spherical
Avocado [30]	Polymer precipitation	51–150 (avg. 99.58 ± 5.09)	−17 ± 1.91	Spherical
Purslane [31]	Sucrose density gradient centrifugation	30–400 (avg. 180)	−31.4	Spherical
Pomegranate [32]	Ultracentrifugation	avg. 152.8	—	Spherical
Chili pepper [33]	Sucrose density gradient centrifugation	50–100	—	Round
Kiwifruit [34]	Polymer precipitation	avg. 35.6 ± 1.16	—	Spherical
Cranberry [35]	Sucrose density gradient centrifugation	80–240	−2.989 ± 0.757	Spherical
Lemon [36]	Sucrose density gradient centrifugation	100	—	Spherical
Lemon [37]	Ultracentrifugation	65 ± 2.7	—	Spherical
Lemon [11]	Electrodialysis	100–400	—	Spherical
Kale [38]	Ultracentrifugation	avg. 212 ± 4.9	—	Spherical
Orange [39]	Ultracentrifugation	74.32 ± 8.75	−21.84 ± 4.84	Round
Orange [39]	Tangential Flow Filtration	76.48 ± 9.42	−23.23 ± 3.62	Round
*Opuntia ficus-indica* [40]	Ultracentrifugation	avg. 161.7	−20.9 ± 6.34	Spherical

## 4. Compositional Analysis

FV-ELNs are enriched with proteins, lipids, nucleic acids, and small-molecule bioactive compounds, the identification of which can be achieved through techniques such as liquid chromatography-tandem mass spectrometry (LC-MS/MS) and various omics approaches.

### 4.1. Proteins

FV-ELNs contain a diverse array of proteins, the composition of which is currently elucidated primarily through sodium dodecyl sulfate-polyacrylamide gel electrophoresis, proteomics, and LC-MS/MS. For instance, label-free quantitative shotgun proteomic analysis was performed on micro- and nanovesicle fractions isolated from four *Citrus* species. Around 600–800 proteins were identified within each individual sample, while a total of 1700 proteins were quantified across all eight analyzed samples (four species, each comprising microvesicle and nanovesicle fractions) [41]. LC-MS/MS-based proteomic profiling of vesicles from grape juice led to the identification of 246 peptides, corresponding to 121 functional proteins, which encompass multiple functional categories including membrane-associated proteins, vesicle trafficking-related proteins, and various functional enzymes [42].

### 4.2. Lipids

Lipids constitute the core components of the phospholipid bilayer membrane of FV-ELNs and serve as signaling molecules that mediate vesicle recognition and uptake by target cells. Currently, the qualitative and quantitative analysis of lipid components in FV-ELNs is primarily performed using thin-layer chromatography and lipidomics. The lipid composition of FV-ELNs is predominantly characterized by phospholipids, mainly including phosphatidylethanolamine (PE), phosphatidic acid (PA), and phosphatidylcholine (PC), along with plant-specific lipids such as digalactosyl–diacylglycerol (DGDG) and monogalactosyldiacylglycerol (MGDG). In grape-derived exosome-like nanovesicles, PA and PE are the most abundant lipid species [23]. For GELNs, PA, DGDG, and MGDG represent its major lipid classes [43].

### 4.3. Nucleic Acids

FV-ELNs contain a variety of nucleic acid components, primarily including microRNA (miRNA), DNA, and other non-coding RNAs, which can be identified and analyzed through techniques such as Northern blotting, real-time quantitative polymerase chain reaction, isotopic labeling, and gene sequencing. Omics-based sequencing studies have revealed that FV-ELNs are enriched in abundant miRNAs. A total of 418 miRNAs were identified from exosome-like nanovesicles derived from 11 edible fruits and vegetables, with 32-127 miRNA species detected for each individual plant source. Bioinformatic target-gene prediction suggests that potential target genes of these miRNAs are enriched in inflammation- and tumor-related signaling pathways, indicating putative biological-regulatory potential. Nevertheless, these findings are largely computational predictions; only a small subset of miRNAs has been validated in vitro, and in vivo cross-kingdom regulatory effects remain highly debated [44]. Gel electrophoresis and sequencing analysis have confirmed that FV-ELNs, including carrot, grape, grapefruit, and ginger, stably harbor miRNA components [45]. In addition to miRNAs, FV-ELNs also carry functional lncRNAs. In vitro experiments using lemon-derived exosome-like nanovesicles (LELNs) have demonstrated that plant lncRNAs can be protected by the vesicles and delivered across kingdoms into human cells, where they regulate host miRNAs through a molecular sponge mechanism. However, in vivo evidence supporting this cross-kingdom regulatory effect remains lacking [46].

### 4.4. Small-Molecule Bioactive Compounds

Vesicles derived from different plant sources contain characteristic bioactive compounds, which are commonly detected using LC-MS/MS and metabolomics. In GELNs, shogaol has been identified [43]. Broccoli-derived exosome-like nanovesicles are rich in sulforaphane [26]. Exosome-like nanovesicles from *Citrus* species are typically abundant in flavonoids such as naringin, among which grapefruit-derived nanovesicles (GDNs) have been found to contain not only naringin but also organic acids including citric acid and glycolic acid [47].

## 5. Biological Activities

**Figure 3:**
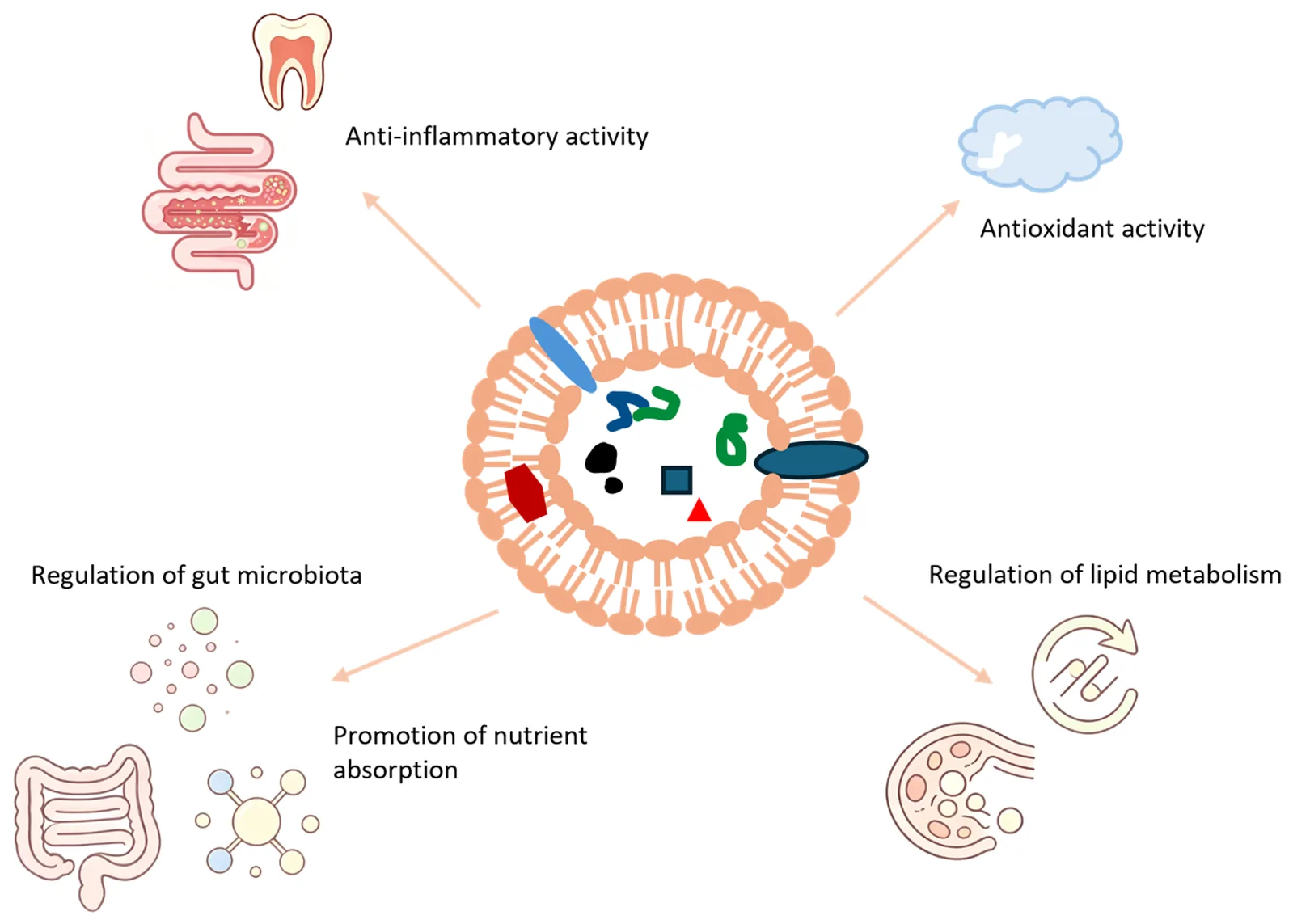
Biological activities of FV-ELNs.

FV-ELNs are enriched with proteins, lipids, nucleic acids, and a variety of small-molecule bioactive compounds. Numerous studies have confirmed that they possess a broad spectrum of biological activities, encompassing anti-inflammatory and gut microbiota-modulating effects, among others. The major biological activities of FV-ELNs are schematically summarized in Figure 3.

To date, investigations into the bioactivity of FV-ELNs have been predominantly conducted at two levels: in vitro cell-based assays and in vivo animal models. At the cellular level, cytotoxicity is typically evaluated via CCK-8 or MTT assays to establish a safe concentration range for subsequent functional studies. Depending on the targeted activity, appropriate in vitro models are employed: hydrogen peroxide or rotenone for inducing oxidative stress, lipopolysaccharide (LPS) for triggering inflammatory responses, and probiotic co-culture systems for assessing gut microbiota modulation, with detection parameters varying according to the activity type. In vivo, commonly utilized animal models include dextran sulfate sodium (DSS)-induced colitis models, high-fat diet (HFD) or high-fat high-sucrose diet (HFHSD)-induced metabolic disorder models, and *Porphyromonas gingivalis* (*P. gingivalis*) infection-induced periodontitis models. Oral gavage represents the predominant route of administration, although local injection and ad libitum consumption in drinking water are also employed in certain contexts. Dosing regimens generally consist of daily or every-other-day administration, with acute models lasting 7 to 14 days and chronic or metabolic models extending to 4 to 12 weeks. Notably, the dosage units of FV-ELNs vary considerably across studies, with some expressed as total protein content (micrograms or milligrams), others as particle number (particles per milliliter), and still others as body weight-based metrics (milligrams per kilogram). The major biological activities and underlying mechanisms of FV-ELNs are summarized in Table 4.

**Table 4:** Biological activities and mechanisms of FV-ELNs.

Biological Activity	Plant Source	Model	Mechanism of Action	Evidence Level	Dose	Duration
Anti-inflammatory	Ginger [43]	DSS	TNF-α, IL-6, IL-1β ↓; IL-10, IL-22 ↑	Animal	0.3 mg/mouse (oral)	7 days
Anti-inflammatory	Ginger [48]	Periodontitis model	TNF-α, IL-6, IL-1β, bone loss, osteoclast ↓; osteoblast ↑	Animal	4.0 × 10^8^ particles/mL (in drinking water, ad libitum)	42 days
Anti-inflammatory	Ginger [6]	Periodontitis model	NF-κB, ROS, TNF-α, IL-6, IL-1β ↓; CAT, T-AOC ↑	Animal	100 μg/mL, 25 μL/site, local injection (every other day)	14 days
Anti-inflammatory	Grape [23]	DSS	Lgr5^+^ intestinal stem cells ↑	Animal	2 mg/mouse (oral)	7 days
Anti-inflammatory	Grape [24]	Acetic-acid	TNF-α, IL-1β, IL-2, IL-6, IL-8, IFN-γ ↓; IL-10 ↑	Animal	10 mg protein/rat/d, topical mucosa	6 days
Anti-inflammatory	*Opuntia ficus-indica* [40]	LPS	IL-1β, TNF-α ↓; IL-4, IL-10 ↑	In vitro	5–25 μg/mL	24 h
Anti-inflammatory	Grapefruit [49]	DSS	TNF-α, IL-6, IL-1β ↓; HO-1, IL-10 ↑	Animal	2 mg/kg (oral)	7 days
Anti-inflammatory	Broccoli [26]	DSS; T cell transfer	IL-10 ↑; IFN-γ, IL-17A, TNF-α ↓; IL-10 ↑	Animal	250 μg/mouse (oral)	22 days
Anti-inflammatory	Tangerine (cv. ‘Dahongpao’) [50]	LPS	NO, TNF-α, IL-6, IL-1β ↓	In vitro	400 μg/mL	24 h
Gut microbiota regulation	Lemon [51]	*L. rhamnosus* GG	Msp1, Msp3 ↓; intestinal lactobacilli ↑	In vitro	1 × 10^10^ particles/mL; 5 × 10^10^ particles/mL	6–12 h
		Mouse gut	*Lactobacillus* ↑; bile resistance ↑	Animal	5 × 10^9^ particles/g bodyweight (twice daily, oral gavage)	7 days
Regulation of intestinal zinc absorption and transport	Tomato [52]	Differentiated Caco-2; HIEC-6	MT1X, MT1G, MT2A, metallothioneins, intracellular zinc ↓; basolateral zinc secretion ↑	In vitro	1 μg total protein content	24 h
Antioxidant	Carrot [27]	H_2_O_2_; 6-OHDA	ROS, Caspase-3 activity ↓; Nrf2, HO-1, NQO-1 ↑	In vitro	1.0 × 10^11^ particles/mL	24 h
Antioxidant	Blueberry [19]	Rotenone	ROS ↓	In vitro	50–200 μg/mL	4 h
Antioxidant	*Opuntia ficus-indica* [40]	TBH	ROS ↓	In vitro	5–25 μg/mL	2 h
Antioxidant	Tangerine (cv. ‘Dahongpao’) [50]	H_2_O_2_	MDA ↓; SOD, T-AOC ↑	In vitro	400 μg/mL	24 h
Regulation of lipid metabolism	Orange [53]	HFHSD	FABP2, FATP4, MTP ↓; ANGPTL4, ACAT2, DGAT1, villus length ↑	Animal	150 μg/mouse (oral)	4 weeks
		Caco-2/HT29-MTX	Intestinal TG uptake ↑	In vitro	1 μg/mL	3 h
Regulation of lipid metabolism	Pomegranate [32]	MASLD	MMP2 ↓; ZO-1 ↑	Animal	1 mg/kg/day	5 weeks
Regulation of lipid metabolism	Blueberry [19]	HFD	FAS, ACC1, TC, TG, LDL, ALT, AST ↓; HDL ↑	Animal	100 mg/kg	4 weeks

MT1X: Metallothionein 1X, MT1G: Metallothionein 1G, MT2A: Metallothionein 2A, TBH: tert-Butyl hydroperoxide, NQO-1: NAD (P) H:quinone oxidoreductase 1, T-AOC: Total Antioxidant Capacity, FABP2: Fatty acid-binding protein 2, FATP4: Fatty acid transport protein 4, MTP: Microsomal triglyceride transfer protein, ANGPTL4: Angiopoietin-like protein 4, ACAT2: Acyl-CoA cholesterol acyltransferase 2, DGAT1: Diacylglycerol acyltransferase 1; MMP2: Matrix Metalloproteinase 2, FAS: Fatty Acid Synthase, ACC1: Acetyl-CoA Carboxylase 1, TC: Total Cholesterol, TG: Triglycerides, LDL: Low-Density Lipoprotein, ALT: Alanine Aminotransferase, AST: Aspartate Aminotransferase, HDL: High-Density Lipoprotein; “↑” represents increase; “↓” represents decrease.

Of note, most of the currently reported bioactivity studies have been performed using intact FV-ELNs. However, these samples may contain co-isolated phenolic compounds, proteins, and other bioactive impurities that could contribute to or interfere with the observed effects. Therefore, the extent to which the reported activities are attributable to the vesicles themselves, rather than to co-purified contaminants, remains to be fully elucidated. Future studies should employ more rigorous purification strategies and orthogonal characterization approaches to better distinguish vesicle-specific effects from those arising from co-isolated components, thereby enabling a more accurate assessment of the intrinsic bioactivities of FV-ELNs.

### 5.1. Anti-Inflammatory Activity

Chronic low-grade inflammation in the body is a critical predisposing factor for intestinal damage, oral lesions, and systemic metabolic disorders. FV-ELNs can modulate classical signaling pathways such as nuclear factor-κB (NF-κB) and the wingless/integrated β-catenin (Wnt/β-catenin) pathway, thereby balancing the secretion of inflammatory cytokines, regulating the differentiation of mucosal immune cells, and protecting mucosal tissues.

#### 5.1.1. Alleviation of Intestinal Inflammation

The intestinal mucosa serves as a crucial immunological barrier in the body. Persistent low-grade intestinal inflammation can readily lead to mucosal damage, impaired nutrient absorption, and gut microbiota dysbiosis. A variety of FV-ELNs have been shown to effectively downregulate the expression of pro-inflammatory cytokines including tumor necrosis factor-α (TNF-α), interleukin-1β (IL-1β), interleukin-6 (IL-6), interferon-γ (IFN-γ), and interleukin-17A (IL-17A), while concurrently promoting the secretion of anti-inflammatory cytokines such as interleukin-10 (IL-10) and interleukin-22 (IL-22), thereby reshaping the anti-inflammatory immune microenvironment of the gut. Among these, GELNs alleviate inflammatory injury and promote mucosal repair by down-regulating pro-inflammatory cytokines and elevating anti-inflammatory mediators in the intestine [43]. Grape-derived exosome-like nanovesicles activate the Wnt/β-catenin signaling pathway, upregulate the expression of repair-related genes including axis inhibition protein 2, cyclin D1, and cellular myelocytomatosis oncogene, and promote the proliferation of Lgr5^+^ intestinal stem cells and epithelial cell regeneration, thereby achieving remodeling and repair of the intestinal barrier [23]. GDNs target intestinal macrophages and may ameliorate DSS-induced colonic inflammatory injury through upregulation of heme oxygenase-1 (HO-1) and IL-10 expression [49]. Furthermore, broccoli-derived nanoparticles induce the generation of tolerogenic dendritic cells, suppress excessive inflammatory responses, and continuously improve chronic intestinal inflammatory conditions [26].

#### 5.1.2. Treatment of Periodontitis

Chronic periodontal inflammation not only causes damage to gingival tissues but can also induce systemic persistent low-grade inflammation through the release of inflammatory cytokines into circulation and ectopic migration of pathogenic bacteria. GELNs exhibit significant inhibitory effects against *P. gingivalis*. The unsaturated PA encapsulated within GELNs specifically binds to hemin-binding protein 35 on the bacterial surface, which mediates selective internalization of GELNs into bacteria and subsequent suppression of bacterial proliferation [48]. Concurrently, GELNs suppress the activation of the NF-κB signaling pathway, alleviate local oxidative stress and inflammatory responses, and promote the proliferation and migration of periodontal ligament fibroblasts, facilitating the repair of tissue damage caused by periodontitis [6].

### 5.2. Regulation of Gut Microbiota and Promotion of Nutrient Absorption

In addition to alleviating intestinal inflammation and repairing the mucosal barrier, FV-ELNs further maintain and consolidate intestinal homeostasis through modulation of gut microbiota structure and promotion of mineral absorption. LELNs are rich in galacturonic acid-type pectin polysaccharides, which activate ribonuclease P of *Lactobacillus rhamnosus* GG (*L. rhamnosus* GG) to mediate the specific degradation of tRNA and other transfer RNAs, thereby inhibiting the translation of major secreted protein 1/3 (Msp1/3) in lactic acid bacteria and significantly enhancing their bile tolerance. Animal studies have demonstrated that LELNs effectively increase the abundance of lactic acid bacteria in the mouse small intestine and stabilize the gut microecological structure [51]. Furthermore, tomato-derived exosome-like nanovesicles downregulate the expression of metallothionein family genes, reduce intracellular zinc chelation levels, and promote the basolateral secretion of zinc ions by differentiated intestinal epithelial cells, thereby effectively improving the bioavailability of zinc in the body and contributing to the maintenance of intestinal functional homeostasis from the perspective of nutrient absorption [52].

### 5.3. Antioxidant Activity

Oxidative stress arises when the equilibrium between intracellular free radical levels and the antioxidant defense system is disrupted. Elevated oxidative stress triggers apoptosis and ultimately leads to cell death. FV-ELNs encapsulate a variety of small RNAs, vitamin C, micronutrients, and bioactive small molecules, and accumulating evidence has demonstrated that numerous FV-ELNs exhibit excellent antioxidant activity in vitro. Among these, FV-ELNs derived from ginger, mushroom, lemon, and onion possess prominent free radical scavenging capacity, while those from green pepper, lemon, yam, and broccoli effectively inhibit oxidative stress-induced apoptosis [54]. FV-ELNs from Tangerine (cv. ‘Dahongpao’) are rich in bioactive compounds such as total phenols and exert antioxidant effects through a dual mechanism: on one hand, they directly scavenge excessive free radicals to alleviate oxidative damage; on the other hand, they significantly enhance the activities of superoxide dismutase (SOD) and catalase (CAT) in H_2_O_2_-damaged RAW264.7 cells, thereby augmenting the overall cellular antioxidant capacity, while concurrently reducing the accumulation of oxidative damage markers including malondialdehyde (MDA) and nitric oxide (NO) [50]. Carrot-derived FV-ELNs activate the nuclear factor erythroid 2-related factor 2 (Nrf2) signaling pathway, effectively downregulating intracellular reactive oxygen species (ROS) levels and caspase-3 activation, thereby attenuating oxidative stress-mediated apoptosis [27].

### 5.4. Regulation of Lipid Metabolism

FV-ELNs can effectively modulate lipid metabolism and ameliorate metabolic disorders, exerting positive effects on blood lipid regulation and reducing fat accumulation. Orange-derived exosome-like nanovesicles regulate key genes involved in lipid metabolism, inhibit fatty acid absorption and chylomicron synthesis, while concurrently promoting hepatic triglyceride release and transport, thereby reducing fat deposition in the intestine and liver [53]. A small-scale open-label human pilot study reported that healthy subjects administered a formulation containing LELNs and *citrus* flavonoids exhibited reduced serum low-density lipoprotein cholesterol (LDL-C) levels. However, given the uncontrolled design of this study and the complexity of the test formulation, the individual contribution of LELNs to the observed effects cannot be clearly delineated. Consequently, current evidence is insufficient to conclude that LELNs constitute a safe and effective dietary intervention for dyslipidemia. Well-designed randomized controlled trials are warranted to confirm the specific role of LELNs in lipid metabolism regulation and to establish their safety profile as a dietary intervention [55]. Pomegranate-derived exosome-like nanovesicles upregulate the expression of ZO-1 and Occludin while simultaneously inhibiting oxidative stress and fibrosis-related proteins such as matrix metalloproteinase 2 (MMP2), thereby reducing hepatic lipid deposition and oxidative damage, and maintaining metabolic homeostasis [32].

FV-ELNs represent an emerging class of natural nanovesicles that exhibit diverse biological activities, including anti-inflammatory, antioxidant, gut microbiota-modulating, and lipid metabolism-regulatory effects. Although substantial evidence from in vitro and animal studies has been accumulated regarding their bioactivity, human investigations remain exceedingly scarce, and the translational gap between preclinical findings and clinical applications remains to be bridged.

## 6. Application Potential, Bottlenecks and Prospects of FV-ELNs in the Food Sector

With the growing public awareness of health, functional foods have become a research hotspot. FV-ELNs have attracted considerable attention in the field of food science due to their health benefits and multifunctionality. These nanovesicles offer the dual potential to enhance the nutritional and health-promoting properties of foods while serving as natural agents for extending shelf life. However, current research on FV-ELNs remains predominantly confined to in vitro cell culture systems and rodent models, with no human interventional trials or pilot-scale food matrix studies reported to date. The translational gap between preclinical findings and validated food-industry applications remains substantial. Nonetheless, the available evidence, particularly the favorable gastrointestinal stability and multifunctional bioactivities of FV-ELNs, provides a scientific foundation for exploring their potential applications in food preservation, functional food development, and bioactive compound delivery.

### 6.1. Application Potential of FV-ELNs in the Food Sector

#### 6.1.1. FV-ELNs as Natural Antioxidants and Green Food Preservatives

At present, the food industry predominantly employs synthetic antioxidants such as butylated hydroxytoluene and butylated hydroxyanisole for food preservation and shelf-life extension. These synthetic additives offer high antioxidant efficiency and stable preservation effects, effectively delaying oxidative deterioration and prolonging shelf life. However, long-term excessive intake may impose a burden on metabolic processes and pose potential food safety risks. With increasing consumer health awareness, natural, additive-free, and clean-label products have become a prevailing trend in food consumption. The development of novel green preservation materials that are safe, non-toxic, stable in performance, and derived from natural sources has thus emerged as a research priority in the food processing field.

FV-ELNs are naturally enriched with a variety of small-molecule bioactive compounds, including polyphenols, flavonoids, and terpenoids, and exhibit strong free radical scavenging capacity and antioxidant potential in vitro, making them promising natural antioxidant candidates for future food applications. For instance, blueberry-derived exosome-like nanovesicles have been shown to possess antioxidant properties [19], suggesting their potential for incorporation into fruit juices or further development into fortified functional beverages. In the field of food packaging materials, the intrinsic antibacterial and antioxidant activities of FV-ELNs offer potential for extending product shelf life. While research on the direct application of FV-ELNs as food additives or preservatives remains limited, studies on exosomes from other sources can provide useful insights. For example, food storage simulation experiments have demonstrated that mushroom-derived exosome-like nanovesicles significantly inhibit lipid peroxidation and protein oxidative denaturation in chicken sausages during storage, effectively delaying flavor deterioration and quality degradation, thereby markedly improving food storage stability [56].

#### 6.1.2. FV-ELNs as Gut Health-Modulating Functional Foods

In vitro studies have confirmed that FV-ELNs maintain structural integrity under simulated gastrointestinal conditions, supporting their feasibility as food ingredients. For example, grape-derived exosome-like nanovesicles withstand sequential digestion by pepsin and pancreatin-bile extract [23], while ginger-derived exosome-like nanovesicles remain intact when exposed to simulated gastric fluid (pH 2.0, containing pepsin) followed by transfer to simulated intestinal fluid (pH 6.5, containing bile extract and pancreatin) [43]. This gastrointestinal stability provides the prerequisite for FV-ELNs to exert their bioactivities in the intestinal tract. FV-ELNs possess anti-inflammatory and gut microbiota-regulating bioactivities, enabling them to repair damaged intestinal mucosa and balance the gut microecology. Grape-derived exosome-like nanovesicles induce the proliferation of Lgr5^+^ intestinal stem cells, activate the Wnt/β-catenin signaling pathway, accelerate intestinal mucosal epithelial regeneration and structural reconstruction, and effectively protect mice from DSS-induced colitis [23]. *Houttuynia cordata*-derived exosome-like nanovesicles specifically target inflamed colonic tissues, reduce the abundance of harmful bacteria, increase the proportion of beneficial bacteria, and alleviate colitis symptoms [57]. FV-ELNs show promise as functional ingredients for gut health-oriented products, with potential applications in the development of nutritional interventions for individuals with irritable bowel syndrome, inflammatory bowel disease in remission, and populations with gastrointestinal sensitivity.

#### 6.1.3. FV-ELNs as Delivery Vehicles for Functional Bioactive Compounds

FV-ELNs can also serve as delivery vehicles for natural bioactive compounds. Leveraging their drug-loading properties enables the encapsulation of polyphenols [58], curcumin [59], and other bioactive substances, thereby enhancing their stability and bioavailability. Studies have demonstrated that *Lentinula edodes*-derived exosome-like nanovesicles can efficiently encapsulate curcumin, improving its stability during simulated gastrointestinal digestion, prolonging small intestinal retention time, and promoting its metabolic conversion through interactions with beneficial gut microbiota [60]. Similarly, *Spinacia oleracea*-derived exosome-like nanovesicles encapsulating curcumin remain stable in saliva and gastric acid, achieve sustained release in intestinal fluid, and effectively alleviate neuroinflammatory damage [61]. These findings indicate that FV-ELNs can exhibit favorable gastrointestinal tolerance and release behavior upon encapsulation of different bioactive compounds. However, such properties are highly source- and formulation-dependent. Differences in lipid composition, surface charge, and endogenous cargo among vesicles from different plant sources may result in substantial variations in their ability to traverse the gastrointestinal barrier and in their release kinetics. The loading strategy, whether encapsulation within the lumen, membrane incorporation, or surface adsorption, and the formulation type, including freshly prepared, lyophilized, or surface-modified variants, also significantly influence their stability and release profiles [62,63]. Furthermore, although FV-ELNs possess certain advantages as natural delivery systems, including biocompatibility, low immunogenicity, and the ability to cross biological barriers, direct comparative studies with established food delivery platforms such as liposomes, nanoemulsions, and biopolymer nanoparticles are currently lacking. Future research should prioritize such systematic comparative studies to objectively evaluate the relative advantages and limitations of FV-ELNs as delivery vehicles for functional food applications.

### 6.2. Core Bottlenecks in the Industrial Translation of FV-ELNs

#### 6.2.1. Isolation Methods Require Optimization

Ultracentrifugation, historically regarded as the conventional reference method for exosome isolation, is operationally simple but suffers from low yield, particle aggregation, and co-isolation of contaminants with similar sedimentation coefficients, rendering it suitable primarily for basic mechanistic studies rather than industrial-scale production [64]. Although modified approaches such as tangential flow filtration and chromatographic purification can improve vesicle purity and preserve bioactivity, they are associated with high equipment costs [65]. Furthermore, factors including cultivation region, harvest season, and maturity stage of plant materials can lead to batch-to-batch variations in particle size and bioactive component content of FV-ELNs. Studies have confirmed that GELNs harvested in July exhibit superior colloidal stability and smaller hydrodynamic diameter compared to those harvested in other months, indicating that seasonal variation significantly influences the physicochemical properties of FV-ELNs [66]. To date, no standardized isolation parameters have been established within the industry, making it difficult to ensure product consistency across different batches.

#### 6.2.2. Storage and Transportation Technologies Require Improvement

FV-ELNs are prone to aggregation, structural disruption, and leakage of internal bioactive components under ambient conditions, exhibiting overall poor storage stability, which poses considerable challenges for their preservation and storage. Relevant stability studies have demonstrated that exosome-like nanovesicles derived from *Kaempferia parviflora* undergo rapid lipid degradation at 4 °C and room temperature, while maintaining structural and functional stability only under ultra-low temperature conditions of −20 °C and −80 °C, with a stable preservation period of up to 8 weeks [67]. At present, laboratory-scale research predominantly relies on ultra-low temperature cryopreservation to maximize the retention of FV-ELN bioactivity. However, this preservation approach is costly and demands stringent conditions, rendering it incompatible with the requirements of industrial food production, storage, and transportation. Currently, there remains a notable deficiency in industry-oriented research on food-grade FV-ELNs. Systematic studies on lyophilization process optimization and long-term storage stability at ambient temperatures are scarce, and few investigations have examined the effects of conventional food processing techniques, such as pasteurization and high-temperature short-time sterilization, on the microstructure and bioactivity of FV-ELNs. These knowledge gaps significantly impede their large-scale application in the food industry.

#### 6.2.3. Quality Control and Safety Systems Require Improvement

Although FV-ELNs exhibit promising biological activities and considerable application potential, their safety for food applications and the underlying mechanisms of action remain to be fully elucidated. To date, the industry has not yet established unified quality control standards for FV-ELNs, lacking clearly defined parameters for particle size distribution, impurity limits, detection protocols for hazardous substances, and standardized bioactivity evaluation systems. At the mechanistic level, current research predominantly focuses on the phenotypic regulatory effects of FV-ELNs, whereas the interaction patterns between FV-ELNs and intestinal cells or immune cells upon entry into the body, as well as the in vivo metabolic fate of the intact vesicular components, remain poorly understood. Furthermore, sufficient data supporting their long-term food safety are currently unavailable. The potential risks of FV-ELNs, including cytotoxicity, in vivo bioaccumulation, chronic immune stress, and metabolic disorders, have yet to be systematically verified. In addition, the currently reported bioactivities may be partly attributed to co-isolated phenolic compounds, proteins, and other impurities rather than to the vesicles themselves, underscoring the need for more rigorous purification and orthogonal characterization in future studies.

### 6.3. Future Perspectives

The translation of FV-ELNs from laboratory research to industrial application still faces substantial challenges. First, isolation processes require optimization to improve raw material utilization efficiency, while standardized isolation parameters tailored to different raw materials should be established to minimize batch-to-batch product variability. Second, formulation technologies such as microencapsulation and lyophilized formulations compatible with FV-ELNs need to be developed to facilitate ambient-temperature storage; meanwhile, the effects of various sterilization methods and processing techniques on their structural integrity and bioactivity should be quantified to define safe processing thresholds. Third, unified evaluation criteria should be established covering key parameters including particle size, concentration, zeta potential, purity, and bioactivity; long-term oral toxicity, allergenicity, and nanobioaccumulation data need to be supplemented through toxicological studies, and the underlying mechanisms of action warrant in-depth investigation. With continued advances in isolation processes and formulation technologies, coupled with the progressive refinement of quality control and safety systems, FV-ELNs are expected to emerge as a novel class of natural functional ingredients for the food industry, offering substantial research and development value and considerable potential for industrial translation.

## 7. Conclusions

FV-ELNs are nanoscale vesicles derived from fruit and vegetable tissues, containing proteins, nucleic acids, lipids, and small-molecule bioactive compounds. They are characterized by wide availability, favorable safety, and good structural stability. With antioxidant, anti-inflammatory, and gut microbiota-modulating properties, FV-ELNs can be used as natural functional agents for food preservation and functional food development. They also serve as carriers for bioactive compounds, encapsulating ingredients like curcumin and probiotics to improve their gastrointestinal stability and oral bioavailability. Despite multiple challenges in industrial application, continued research is expected to facilitate their use in natural preservatives, functional foods, and bioactive compound delivery. Ultimately, FV-ELNs may contribute to the green and functional transformation of the food industry, support the efficient use of agricultural and forestry resources, and promote the upgrading of the health-oriented food sector.

## Figures and Tables

**Table 1:** Advantages, disadvantages and applicable scenarios of FV-ELNs isolation techniques.

Isolation Method	Advantages	Disadvantages	Applicable Scenarios
Ultracentrifugation	Simple operation; well-established protocol	Time-consuming; low single-batch processing capacity	Small-scale laboratory applications
Sucrose density gradient centrifugation	High purity	Cumbersome procedure; time-consuming	Basic research requiring high purity
Polymer precipitation	Simple operation; no sophisticated equipment required	Low purity	Large-scale crude extraction
Electrodialysis	Cost-effective; high separation efficiency	Cumbersome procedure; requires periodic reversal of electric field direction	Rapid and efficient acquisition
Ultrafiltration	Fast processing; scalable	Membrane susceptible to clogging	Large-scale isolation
Size exclusion chromatography	Preserves structural integrity; mild conditions	High equipment cost	High-purity applications; activity preservation required
Immunoaffinity isolation	High selectivity	Lack of well-characterized markers	Isolation of specific vesicle subpopulations

**Table 2:** Advantages and disadvantages of morphological characterization techniques for FV-ELNs.

Characterization Method	Advantages	Disadvantages
TEM	High resolution; enables observation of fine internal structures	Requires vacuum and dehydration fixation; prone to sample deformation
AFM	Non-destructive; high resolution	Cumbersome operation; demanding requirements; slow scanning speed; high cost
SEM	Suitable for surface morphology and three-dimensional structure observation	Low resolution; difficult to achieve fine characterization
Cryo-EM	No extensive processing required; results closely reflect native state	Expensive equipment; complex procedures; limited application

## Data Availability

No new data were created or analyzed in this study. Data sharing is not applicable to this article.
